# Patient and practice level factors associated with seasonal influenza vaccine uptake among at-risk adults in England, 2011 to 2016: An age-stratified retrospective cohort study

**DOI:** 10.1016/j.jvacx.2020.100054

**Published:** 2020-01-13

**Authors:** Matthew M. Loiacono, Salaheddin M. Mahmud, Ayman Chit, Robertus van Aalst, Jeffrey C. Kwong, Nicholas Mitsakakis, Luke Skinner, Edward Thommes, Hélène Bricout, Paul Grootendorst

**Affiliations:** a Leslie Dan Faculty of Pharmacy, University of Toronto, 144 College St, Toronto, ON M5S 3M2, Canada; b College of Pharmacy, Rady Faculty of Health Sciences, University of Manitoba, Winnipeg, MB R3E 0T5, Canada; c Vaccine and Drug Evaluation Centre, Max Rady College of Medicine, Rady Faculty of Health Sciences, University of Manitoba, Winnipeg, MB R3E 0T5, Canada; d Vaccine Epidemiology and Modeling, Sanofi Pasteur, 1 Discovery Dr, Swiftwater, PA 18370, United States; e Department of Health Sciences, University Medical Center Groningen, University of Groningen, 9700 AB Groningen, the Netherlands; f ICES, 2075 Bayview Ave, Toronto, ON M4N 3M5, Canada; g Public Health Ontario, 480 University Ave #300, Toronto, ON M5G 1V2, Canada; h Dalla Lana School of Public Health, University of Toronto, 155 College St, Toronto, ON M5T 3M7, Canada; i Department of Family & Community Medicine, University of Toronto, 500 University Ave, Toronto, ON M5G 1V7, Canada; j University Health Network, 101 College St, Toronto, ON M5G 1L7, Canada; k Sanofi Pasteur, 410 Thames Valley Park Dr, Earley, Reading RG6 1RH, United Kingdom; l Department of Mathematics & Statistics, University of Guelph, 50 Stone Road East, Guelph, ON N1G 2W1, Canada; m Sanofi Pasteur, 14 Espace Henry Vallée, 69007 Lyon, France

**Keywords:** Seasonal influenza vaccine, Vaccine uptake, General practice, Determinants, Clinical Practice Research Datalink, CPRD, Clinical Practice Research Datalink, GP, general practitioner, IMD, Index of Multiple Deprivations, NHS, National Health Service, PHE, Public Health England, SES, socioeconomic status, UK, United Kingdom, VE, vaccine effectiveness, SIV, season influenza vaccine, WHO, World Health Organization

## Abstract

We sought to gain insights into the determinants of seasonal influenza vaccine (SIV) uptake by conducting an age-stratified analysis (18–64 and 65+) of factors associated with SIV uptake among at-risk adults registered to English practices. Records for at-risk English adults between 2011 and 2016 were identified using the Clinical Practice Research Datalink database. SIV uptake was assessed annually. The associations of patient, practice, and seasonal characteristics with SIV uptake were assessed via cross-sectional and longitudinal analyses, using mixed-effects and general estimating equation logistic regression models. Overall SIV uptake was 35.3% and 74.0% for adults 18–64 and 65+, respectively. Relative to white patients, black patients were least likely to be vaccinated (OR_18-64_: 0.82 (95% CI: 0.80, 0.85); OR_65+_: 0.59 (95% CI: 0.56, 0.62)), while Asian patients among 18–64 year olds were most likely to be vaccinated (OR_18-64_: 1.10 (95% CI: 1.07, 1.13)). Females were more likely than males to be vaccinated among 18–64 year olds (OR_18-64_: 1.19 (95% CI: 1.18, 1.20)). Greater socioeconomic deprivation was associated with decreased odds of uptake among older patients (OR_65+_: 0.74 (95% CI: 0.71, 0.77)). For each additional at-risk condition, odds of uptake increased (OR_18-64_: 2.33 (95% CI: 2.31, 2.36); OR_65+_: 1.39 (95% CI: 1.38, 1.39)). Odds of uptake were highest among younger patients with diabetes (OR_18-64_: 4.25 (95% CI: 4.18, 4.32)) and older patients with chronic respiratory disease (OR_65+_: 1.60 (95% CI: 1.58, 1.63)), whereas they were lowest among morbidly obese patients of all ages (OR_18-64_: 0.68 (95% CI: 0.67, 0.70); OR_65+_: 0.97 (95% CI: 0.94, 0.99)). Prior influenza season severity and vaccine effectiveness were marginally predictive of uptake. Our age-stratified analysis uncovered SIV uptake disparities by ethnicity, sex, age, socioeconomic deprivation, and co-morbidities, warranting further attention by GPs and policymakers alike.

## Highlights

• Disparities in uptake by ethnicity, varying by age, were evident.
• Older adults with higher socioeconomic deprivation were less likely to be vaccinated.
• Patients with morbid obesity had the lowest odds of being vaccinated.
• Patients who had more annual GP consultations were more likely to be vaccinated.

## 1 Introduction

Influenza is a contagious viral disease that results in up to five million cases of severe illness and 650,000 deaths worldwide each year [1]. Complications from influenza can be particularly severe among children, the elderly, pregnant women, those with certain chronic health conditions, and immunosuppressed individuals [1]. In the United Kingdom (UK), the burden of influenza is similarly significant; each year there are an estimated 18,500–24,800 deaths, 19,000–31,200 hospital admissions, and 779,000–1,164,000 general practitioner (GP) consultations attributable to influenza [2].

The seasonal influenza vaccine (SIV) remains the best tool available to reduce influenza-associated morbidity and mortality. As a result, governments routinely provide price subsidies to promote its use. The National Health Service (NHS) in the UK recommends and offers free SIV to all children aged 2–11 years old, as well as to older children and adults considered to be at-risk for serious influenza complications. Patients are considered to be at-risk if they are over 65 years old, or under 65 with at least one risk factor (e.g. pregnancy, chronic disease, morbid obesity, etc.) [3], [4], [5]. Despite the UK’s high SIV uptake among older adults (65 years of age or older), uptake among younger at-risk adults (18–64) is suboptimal. Further, uptake across the entire population has been stagnant for nearly two decades [6], [7].

Identifying and understanding determinants of vaccine uptake are not only important for designing policies to improve uptake, but these findings may also provide insights as to the underlying drivers of differences in uptake observed across the population. While previous studies of vaccine uptake determinants have demonstrated homogeneity in some uptake determinants across regions, there still remains strong evidence of heterogeneity of determinants between different countries, including socioeconomic status (SES), sex, age, physician visits, and social characteristics [8], [9]. Consequently, insights gained from such studies may not be universally applicable, whereas country-specific studies may generate more applicable findings.

Previous studies have investigated factors associated with SIV uptake [8], [10], [11], [12], [13], [14], but the majority of these studies relied on survey data or small sample sizes, which may introduce errors due to recall, non-response, or selection bias [15], [16]. Large databases of primary care electronic health records, on the other hand, enable researchers to identify and assess larger, potentially more generalizable cohorts of individuals, without relying on self-reported individual responses [17]. In the UK, the Clinical Practice Research Datalink (CPRD), a research service operating within the UK Department of Health, collects and maintains a database of GP records for research purposes. Containing the records of 11 million currently enrolled patients across nearly 600 practices in the UK, CPRD’s database has been used in more than 2,300 peer-reviewed publications [18], [19].

Although the CPRD database has previously been used to investigate SIV uptake in the UK, only one study [12] to our knowledge has investigated said factors among the general population of at-risk individuals via multivariable regression modeling [12], [20], [21]. In their analysis, factors associated with SIV and pandemic influenza vaccine uptake were investigated simultaneously. Given the irregularity of a pandemic influenza season, it stands that their SIV-specific findings may not be directly applicable to non-pandemic seasons. Additionally, their study population consisted of at-risk individuals 0–110 years of age. When interest lies in identifying factors of uptake specifically among at-risk adults, the estimated associations from such an analysis may be biased by the inclusion of non-adults. Similarly, while their analysis also included an age-stratified component, a method useful for uncovering heterogeneity in associations across age, their intermediate age strata combined adolescents (5–17 years old) with younger adults (18–64 years old), potentially rendering these findings susceptible to the same biases.

Building upon prior research and addressing these limitations, we conducted an age-stratified retrospective cohort study (18–64 and 65+ years old) to identify factors associated with SIV uptake among at-risk adults in England, within and across six consecutive non-pandemic influenza seasons, using the CPRD database. The factors investigated encompassed patient demographics, patient-related behaviors and at-risk conditions, patient and practice level SES indicators, and rate of reminders to be vaccinated delivered by the practice. Longitudinally, we assessed the association of the prior influenza season severity and the effectiveness of the prior season’s vaccine with SIV uptake. As a secondary analysis, we investigated the associations between various practice-level characteristics and practice-level SIV uptake.

## 2 Methods

### 2.1 Population and data source

**Fig. 1:**
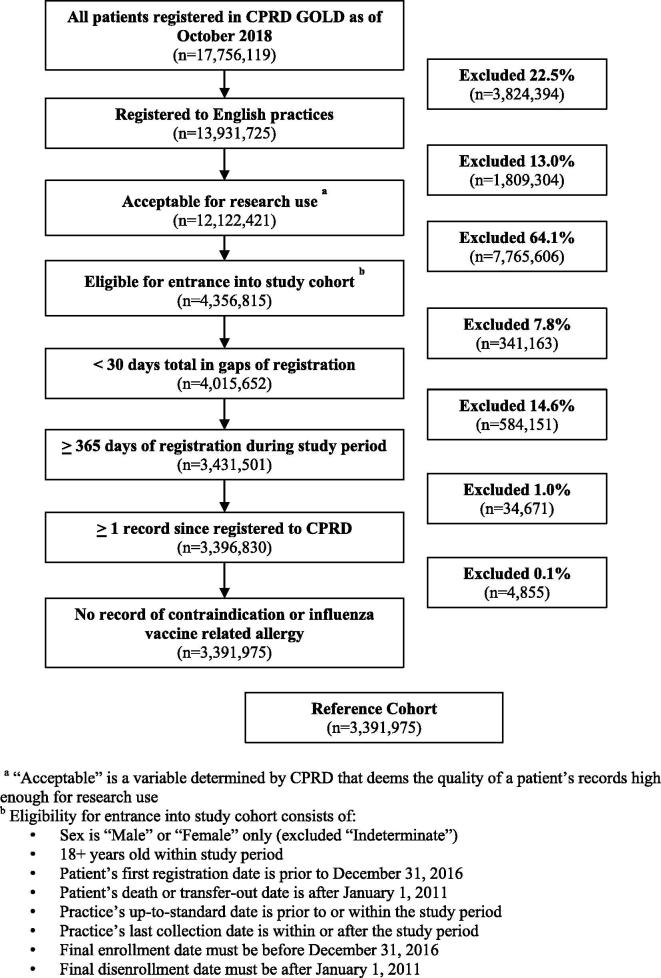
Overview of the selection of the reference cohort.

We constructed a reference cohort (n = 3,391,975) consisting of all adults 18 years or older who were registered to English practices in the CPRD database (CPRD GOLD) for a minimum of 365 consecutive days between January 1, 2011 (study start date) and December 31, 2016 (study end date). The CPRD GOLD version of the CPRD database contains data specifically from practices using Vision software [22]. A minimum registration of 365 days was required to allow us to capture the various patient characteristics of interest. Given that CPRD’s database is considered to be representative of the overall English population, we attempted to preserve this representativeness in the construction of our analytical samples [18]. A detailed overview of the reference cohort selection is provided in Fig. 1. Further details as to the construction of the reference cohort can be found in Supplement 1.

Based on the reference cohort, we constructed annual cohorts of at-risk patients for each year from 2011 to 2016, with each of these further stratified by patient age (18–64 years and 65+ years old). Inclusion in a given annual cohort required a patient to have been enrolled in the reference cohort for the entirety of the specified year (e.g. January 1 to December 31). For those under 65, at least one at-risk condition had to have been present prior to the start of the influenza vaccination campaign (September 1) to be included in an annual cohort, as our study was focused on uptake among at-risk adults.

### 2.2 SIV uptake

The primary outcome measured was patient level SIV uptake. We used Read codes, immunization codes, and product codes to identify all SIVs administered between September 1 and December 31 of each year. For each season, we excluded SIVs administered outside of this window as outliers (<0.5% of patients excluded). All codes used are provided in Supplement 2.

### 2.3 Patient characteristics

Time-varying patient characteristics were determined by the most recent record prior to the start of each season (September 1). Ethnicity was determined based upon the patient’s most recent ethnicity-related Read code in the CPRD database, and was then categorized in accordance to the Office of National Statistics (ONS) Ethnic Groups (e.g. Asian, Black, Mixed, Other, Unknown, or White), with the exception of collapsing the “mixed” and “other” groups for our analysis [23]. SES was measured via the Indices of Multiple Deprivation (IMD 2015), a composite measure of overall deprivation consisting of seven different deprivation domains (income; employment; education, skills, and training; health and disability; crime; barriers to housing and services; and living environment). The IMD is measured at the Lower Layer Super Output Area (LSOA), where LSOA’s are designated to be comparably sized, with an average population size of approximately 1500 residents [24]. The patient IMD data used in this study was acquired via the CPRD data linkage service [25].

Lists of Read codes were used, as arranged by PRIMIS, to identify the following at-risk conditions: pregnancy, chronic renal disease, chronic heart disease, chronic respiratory disease, chronic liver disease, diabetes, immunosuppression, chronic neurological disease, and morbid obesity [23]. PRIMIS is an organization based at University of Nottingham that specializes in the development of GP data extraction methodology, supporting the UK’s national vaccination program [26]. We also calculated a composite risk score as the patient’s total number of at-risk conditions. Annual GP consultations included visits to a GP and GP house calls. All relevant Read codes used can be found in Supplement 2.

### 2.4 Practice characteristics

Practice characteristics included its primary Public Health England (PHE) regional unit (London, Midlands & East, North, or South), urban/rural status as per the ONS’s 2011 Rural Urban classifications, SES measures of the practice area, and rate of reminders to patients to be vaccinated [27]. SES was measured via three small area IMD Domain measures: health deprivation and disability; education, skill and training deprivation; and income deprivation [24]. We opted to investigate individual IMD domains at the practice-level, rather than the composite IMD measure, in hopes of gaining more nuanced insights into practice-level variation in SIV uptake. As for the three specific domains investigated, these were chosen based upon their perceived relevance to SIV uptake, given discussion among the authors as well as prior SIV uptake research [9]. The practice IMD domains data was acquired via the CPRD data linkage service [25]. Practice-level reminders to be vaccinated included records of verbal invitations, phone invitations, mail invitations, or text-message reminders and were identified via Read codes (available in Supplement 2).

### 2.5 Season characteristics

Prior season influenza severity was measured using England-specific surveillance data from the European Centre for Disease Prevention and Control, where season severity was ranked as low, moderate, or high based on the peak of influenza-like-illness consultations [28]. Prior season vaccine effectiveness (VE) was based on estimates of VE reported by PHE and if unavailable, peer-reviewed estimates. Prior season VE was ranked as low (<50%) or high (≥50%). Given the limited number of seasons included in our analysis, relative to the high degree of variability of VE estimates from season to season as well as within seasons, a cutoff of 50% was specified as to minimize potential outlier-induced biases. VE values used and relevant sources are provided in Supplement 3.

### 2.6 Statistical analyses

The cross-sectional analysis of patient and practice level factors used the 2015 season cohorts, to most closely align with the IMD 2015 data. For the longitudinal analysis of patient and seasonal factors, all six annual cohorts were pooled for each age stratum (n = 635,825 18–64 year olds; n = 764,185 65+ year olds).

For the cross-sectional analyses, each predictor’s association with SIV uptake was assessed via bivariate analyses. Predictors with a p-value > 0.2 in the bivariate analyses were excluded from the multivariable analysis. Predictors in the final model with p-value > 0.2 were excluded. Predictor exclusion was negated only if we had evidence suggesting an expected confounding or interaction effect given prior research. Mixed-effects logistic regression models were used, with a random effect for the practice to account for the multi-level nature of the data [29].

For the longitudinal analysis, a multivariable general estimating equation (GEE) logistic regression model was used with clustering specified at the patient level. An exchangeable working correlation matrix was specified after assessing model fit via quasi-AIC (QIC) [30]. Predictors with p-values > 0.2 were iteratively removed from the full longitudinal models. Predictor associations for all analyses were estimated as odds ratios (ORs) with their respective 95% confidence intervals, reported as adjusted ORs from the multivariable analyses. Unadjusted ORs are provided in Supplement 4. For the secondary analysis, mean practice-level SIV uptake rates were tabulated across various quintiles of practice level-factors, as well as assessed visually via scatter plots with smoothed lines of best fit.

All analyses were performed in R 3.4.3 using the *lme4* and *geepack* packages separately for each age stratum [31], [32], [33], excluding the pregnancy variable from the 65+ cohort analyses. This study was approved by the Independent Scientific Advisory Committee (ISAC) of CPRD (Protocol 18_269). Further information about the statistical models used can be found in Supplement 5.

## 3 Results

The reference cohort consisted of 3,391,975 patients who were potentially eligible for inclusion in the annual cohorts, pending at-risk status (Fig. 1). Baseline characteristics of the annual cohorts are presented in Table 1.

**Table 1:** Descriptive statistics for annual cohorts, stratified by age group (18–64 and 65+).

*Variable*	*Season 2011*		*Season 2012*		*Season 2013*		*Season 2014*		*Season 2015*		*Season 2016*	
	*18–64*	*65+*	*18–64*	*65+*	*18–64*	*65+*	*18–64*	*65+*	*18–64*	*65+*	*18–64*	*65+*
	*n = 426805*	*n = 570260*	*n = 416330*	*n = 560772*	*n = 376169*	*n = 514163*	*n = 314631*	*n = 429303*	*n = 223624*	*n = 304658*	*n = 169544*	*n = 212169*
*Sex*												
*Male*	48.8% (208302)	44.7% (254758)	48.8% (203363)	44.9% (252044)	48.9% (184036)	45.1% (232012)	49.0% (154302)	45.3% (194435)	49.2% (109986)	45.3% (138107)	49.2% (83411)	45.5% (96613)
*Female*	51.2% (218503)	55.3% (315502)	51.2% (212967)	55.1% (308728)	51.1% (192133)	54.9% (282151)	51.0% (160329)	54.7% (234868)	50.8% (113638)	54.7% (166551)	50.8% (86133)	54.5% (115556)
*Age (mean ± SD)*	43.4 ± 13.3	75.2 ± 7.7	44.0 ± 13.3	75.0 ± 7.8	44.0 ± 13.2	75.0 ± 7.8	44.1 ± 13.2	75.1 ± 7.8	44.4 ± 13.1	75.1 ± 7.7	44.5 ± 13	75.0 ± 7.6

*Ethnicity*												
*Asian*	3.2% (13510)	1.2% (6953)	3.4% (14079)	1.3% (7348)	3.5% (13273)	1.4% (7056)	3.6% (11308)	1.4% (5821)	3.6% (8063)	1.4% (4410)	4.4% (7543)	1.9% (4072)
*Black*	1.8% (7868)	0.7% (3750)	2% (8527)	0.7% (3942)	2.2% (8208)	0.7% (3776)	2.4% (7550)	0.7% (3120)	2.3% (5124)	0.7% (2077)	2.7% (4550)	0.8% (1753)
*Mixed/Other*	19.9% (84750)	18.9% (107995)	21.0% (87405)	19.8% (110923)	22.3% (83984)	21.1% (108580)	23.1% (72640)	22.5% (96448)	21.7% (48626)	21.0% (63995)	23.3% (39485)	23.2% (49318)
*Unknown*	43.5% (185500)	45.8% (261290)	41.2% (171733)	43.6% (244646)	39.9% (150230)	43.0% (221037)	38.9% (122310)	41.7% (178958)	38.9% (86992)	41.2% (125649)	34.1% (57769)	36.1% (76628)
*White*	31.7% (135177)	33.4% (190272)	32.3% (134586)	34.6% (193913)	32.0% (120474)	33.8% (173714)	32.0% (100823)	33.8% (144956)	33.5% (74819)	35.6% (108527)	35.5% (60197)	37.9% (80398)

*PHE Region*												
*London*	13.2% (56270)	11.0% (62948)	14.4%(59818)	11.9%(66549)	15.5%(58482)	12.8%(65731)	14.3%(44969)	11.5%(49500)	14.5%(32385)	12.1%(36943)	17.3%(29397)	16.2%(34466)
*Midlands & East*	25.2% (107737)	25.0% (142427)	23.9%(99484)	23.8%(133222)	21.7%(81752)	22.2%(114267)	22.0%(69215)	21.7%(93293)	18.9%(42370)	18.3%(55754)	21.8%(36944)	22.6%(47986)
*North*	21.4% (91137)	19.6% (111498)	21.0%(87633)	19.4%(108957)	20.9%(78780)	19.2%(98827)	19.2%(60428)	17.5%(75235)	19.1%(42603)	16.9%(51378)	17.9%(30297)	16.3%(34533)
*South*	40.2% (171661)	44.4% (253387)	40.7% (169395)	44.9% (252044)	41.8% (157155)	45.8% (235338)	44.5% (140019)	49.2% (211275)	47.5% (106266)	52.7% (160583)	43.0% (72906)	44.9% (95184)

*At-Risk Conditions*												
*1* *	85.9% (366564)	32.7% (186555)	85.6% (356472)	32.7% (183121)	85.4% (321218)	32.7% (168263)	85.0% (267365)	32.7% (140209)	84.5% (188953)	32.6% (99182)	84.2% (142782)	32.7% (69341)
*2+*	14.1% (60241)	18.6% (105812)	14.4% (59858)	18.8% (105426)	14.6% (54951)	19.1% (97995)	15.0% (47266)	19.5% (83740)	15.5% (34671)	19.7% (60105)	15.8% (26762)	20.0% (42377)

* Among 65+ patients, 1 at-risk condition indicates an additional at-risk condition beyond the age-based risk.

### 3.1 SIV uptake

Overall SIV uptake was 35.3%, and 74.0% for 18–64 year old patients and 65+ year old patients, respectively (Table 2). Uptake was higher for females than males among the 18–64 year olds, but approximately equivalent between the two sexes among 65+ year olds. Uptake varied across ethnicity, where uptake was lowest among black patients in both age strata, and highest among Asian patients aged 18–64 years old and white patients aged 65+ year olds. Uptake varied across the PHE regions, and was consistently lowest among London-based practices.

**Table 2:** SIV uptake (%) across all seasons 2011–2016 for 18–64 adults and 65+ adults, overall and stratified by sex, ethnicity, region, and at-risk conditions.

*Variable*	*18–64*	*65+*
*Overall*	35.3%	74.0%
*Sex*		
*Male*	33.4%	74.0%
*Female*	37.1%	74.0%
*Ethnicity*		
*Asian*	44.8%	74.8%
*Black*	34.5%	64.3%
*Mixed/Other*	36.3%	76.2%
*Unknown*	31.8%	70.6%
*White*	38.0%	77.1%
*PHE Region*		
*London*	33.8%	70.8%
*Midlands & East*	36.0%	74.7%
*North*	36.8%	76.3%
*South*	34.8%	73.6%
*At-Risk Conditions*		
*Pregnant*	39.8%	–
*Chronic renal disease*	55.1%	83.8%
*Chronic heart disease*	52.7%	84.0%
*Chronic respiratory disease*	28.9%	84.0%
*Chronic liver disease*	41.3%	79.0%
*Diabetes*	69.5%	85.0%
*Immunosuppressed*	48.9%	82.4%
*Chronic neurological disease*	51.3%	80.9%
*Morbid obesity (BMI ≥ 40)*	27.2%	76.5%

(–) There were no pregnancy records among 65+ patients.

**Fig. 2:**
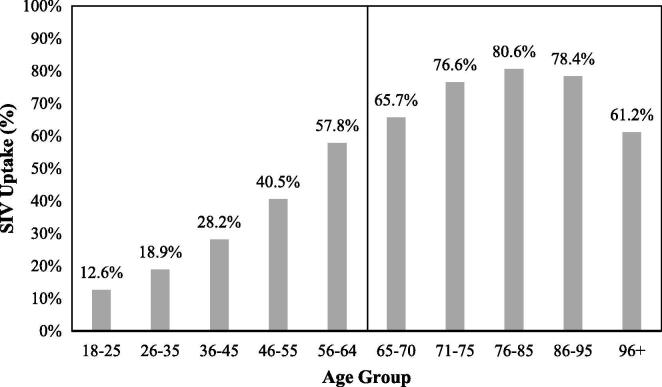
SIV uptake (%) across all seasons 2011–2016 stratified by age groups.

Across at-risk conditions, variability in uptake was considerably greater among 18–64 year olds than 65+ year olds. Notably, uptake was lowest among morbidly obese patients for patients of all ages. Among both strata, uptake was highest for patients with diabetes. Uptake was considerably lower among 18–64 year old patients with chronic respiratory disease relative to other at-risk conditions. With regards to patient age, uptake among the 18–64 year old patients increased monotonically with age, while uptake among the 65+ year old patients only increased with age up to 85 years old, and then decreased (Fig. 2).

### 3.2 Cross-sectional analyses

Results from the cross-sectional analyses are presented in Table 3. Within the 18–64 cohort, sex, ethnicity, smoking status, all at-risk conditions, annual consultations, practice reminders, and practice PHE region were significantly associated with uptake. Odds of uptake were 17% lower for black patients, relative to white patients (OR_adj_ 0.83, 95%_CI_ 0.77, 0.89), but were not significantly different for Asian patients. Relative to non-smokers, smokers were more than 25% less likely to be vaccinated (OR_adj_ 0.73, 95%_CI_ 0.71, 0.75), but for ex-smokers, there was no significant difference in odds of uptake.

**Table 3:** Adjusted odds ratios (OR) from multivariable cross-sectional analyses among patients 18–64 years old and 65+ years old during the 2015–2016 influenza season.

*Variable*		*Season 2015*	
		*Adjusted OR (95% CI)*^a^	
		*18–64*	*65+*
*Sex (female)*		1.09 (1.07, 1.12)*	0.98 (0.96, 1.00)*

*Ethnicity*			
*Asian*		1.04 (0.98, 1.10)	0.89 (0.83, 0.96)*
*Black*		0.83 (0.77, 0.89)*	0.60 (0.55, 0.67)*
*Mixed/Other*		0.96 (0.93, 1.00)*	0.99 (0.96, 1.02)
*Unknown*		0.80 (0.78, 0.82)*	0.71 (0.69, 0.73)*
*White*		Ref.	Ref.

*Patient IMD*			
*1 = Least deprived*		–	Ref.
*2*		–	0.91 (0.88, 0.93)*
*3*		–	0.85 (0.83, 0.88)*
*4*		–	0.80 (0.77, 0.83)*
*5 = Most deprived*		–	0.74 (0.71, 0.77)*
*Unknown*		–	0.84 (0.76, 0.93)*

*Smoking status*			
*Never smoker*		Ref.	Ref.
*Current smoker*		0.73 (0.71, 0.75)*	0.64 (0.63, 0.66)*
*Ex-smoker*		1.02 (1.00, 1.05)	1.11 (1.09, 1.13)*
*Unknown*		0.48 (0.39, 0.58)*	0.28 (0.25, 0.31)*
*Pregnant*		3.06 (2.88, 3.25)*	–b
*Chronic renal disease*		2.22 (2.00, 2.46)*	–
*Chronic heart disease*		1.74 (1.68, 1.80)*	1.36 (1.33, 1.39)*
*Chronic respiratory disease*		1.31 (1.28, 1.35)*	1.53 (1.49, 1.57)*
*Chronic liver disease*		0.85 (0.81, 0.90)*	–
*Diabetes*		3.50 (3.39, 3.61)*	1.48 (1.44, 1.53)*
*Immunosuppression*		2.21 (2.07, 2.35)*	1.23 (1.14, 1.31)*
*Chronic neurological disease*		2.02 (1.94, 2.10)*	1.06 (1.03, 1.09)*
*Morbidly obese (BMI >* *40)*		0.77 (0.75, 0.80)*	0.88 (0.83, 0.94)*
*Unknown*		0.57 (0.54, 0.59)*	0.49 (0.47, 0.52)*
*Composite at-risk score*^c^		1.91 (1.88, 1.96)*	1.33 (1.32, 1.35)*

*GP consultations per year*			
*0–2*		Ref.	Ref.
*3–6*		3.11 (3.01, 3.20)*	2.73 (2.67, 2.80)*
*7+*		6.07 (5.89, 6.25)*	4.41 (4.30, 4.51)*
*% of a practice’s patients reminded to be vaccinated (10% intervals)*		1.02 (1.00, 1.04)*	1.02 (1.01, 1.03)*

*Practice region*			
*London*		Ref.	Ref.
*Midlands & East*		1.32 (1.12, 1.57)*	1.30 (1.11, 1.53)*
*North*		1.19 (1.01, 1.40)*	1.51 (1.29, 1.76)*
*South*		1.22 (1.06, 1.40)*	1.32 (1.15, 1.51)*

(–) variable was excluded due to lack of significance (p > 0.20), unless noted otherwise.

a We adjusted the odds ratios for all characteristics as listed in the table (except for the composite at-risk score, where we omitted the individual at-risk conditions to prevent collinearity). Practice urban/rural status and IMD domain measures were excluded from the model due to lack of significance in the bivariate analyses.

b Pregnancy was excluded from 65+ analysis due to 0 pregnancy records.

c The composite at-risk score was calculated as the patient’s total # of at-risk conditions.

* p < 0.05.

For each additional at-risk condition, odds of uptake increased by approximately 91% (OR_adj_ 1.91, 95%_CI_ 1.88, 1.96). All at-risk conditions were associated with uptake, but only chronic liver disease and morbid obesity were associated with a lower odds of uptake. Patients with diabetes were most likely to be vaccinated (OR_adj_ 3.50, 95%_CI_ 3.39, 3.61). Annual GP consultations were associated with uptake, such that patients with seven or more annual consultations were over six times as likely to be vaccinated, compared to patients with two or fewer consultations (OR_adj_ 6.07, 95%_CI_ 5.89, 6.25). Uptake was least likely among London-based practices. Patient IMD, rurality of the practice, and practice IMD domain measures were not significantly associated with uptake. The variation of the random effect term was indicative of substantial variation in uptake at the practice level (σ^2^ = 0.1255).

Within the 65+ cohort, sex, ethnicity, patient IMD, smoking status, most at-risk conditions, annual consultations, practice reminders, and practice PHE region were significantly associated with uptake (Table 3). Odds of uptake were 40% lower for black patients (OR_adj_ 0.60, 95%_CI_ 0.55, 0.67) and 11% lower for Asian patients (OR_adj_ 0.89, 95%_CI_ 0.83, 0.96), relative to white patients. Socioeconomic deprivation was associated with a lower odds of uptake, such that the most socioeconomically deprived patients (IMD = 5) were 26% less likely to be vaccinated than the least deprived (IMD = 1) (OR_adj_ 0.74, 95%_CI_ 0.71, 0.77). Relative to non-smokers, current smokers were 36% less likely to be vaccinated (OR_adj_ 0.64, 95%_CI_ 0.63, 0.66), while ex-smokers were 11% more likely to be vaccinated (OR_adj_ 1.11, 95%_CI_ 1.09, 1.13).

For each additional at-risk condition, odds of uptake increased by approximately 33% (OR_adj_ 1.33, 95%_CI_ 1.32, 1.35). Chronic renal disease and chronic liver disease were not associated with uptake. All other at-risk conditions were positively associated with uptake, except for morbid obesity (OR_adj_ 0.88, 95%_CI_ 0.83, 0.94). Annual GP consultations were associated with uptake, such that patients with seven or more annual consultations were over four times as likely to be vaccinated, relative to those with two or fewer consultations (OR_adj_ 4.41, 95%_CI_ 4.30, 4.51). Uptake was least likely among London-based practices. Rurality of the practice and practice IMD domain measures were not associated with uptake. Practice level uptake varied substantially, as indicated by the variation of random effect term (σ^2^ = 0.1147).

### 3.3 Longitudinal analyses

Results from the longitudinal analyses are presented in Table 4. The direction and magnitude of the associations overall remained comparable to those from the cross-sectional analyses, with the exception of some characteristics. Among 18–64 year olds, Asian patients were more likely to be vaccinated, compared to white patients (OR_adj_ 1.10, 95%_CI_ 1.07, 1.13). Patients with chronic liver disease were more likely to be vaccinated (OR_adj_ 1.13, 95%_CI_ 1.10, 1.16). Among 65+ year olds, morbidly obese patients remained less likely to be vaccinated, albeit to a lesser degree than observed in the cross-sectional analysis (OR_adj_ 0.97, 95%_CI_ 0.94, 0.99). Longitudinally, uptake decrease across time for both strata. Prior influenza season severity and VE were associated with uptake among 18–64 year old. Among 65+ patients, only prior season VE was associated with uptake.

**Table 4:** Adjusted odds ratios (OR) from multivariate longitudinal analyses among patients 18–64 years old (n = 635,825) and 65+ years old (n = 611,845) from 2011 to 2016.

*Variable*		*Seasons 2011–2016*	
		*Adjusted OR (95% CI)*^a^	
		*18–64*	*65+*
*Sex (female)*		1.19 (1.18, 1.20)*	1.01 (1.00, 1.02)*

*Ethnicity*			
*White*		Ref.	Ref.
*Asian*		1.10 (1.07, 1.13)*	0.85 (0.81, 0.88)*
*Black*		0.82 (0.80, 0.85)*	0.59 (0.56, 0.62)*
*Mixed/Other*		0.95 (0.94, 0.97)*	0.93 (0.92, 0.95)*
*Unknown*		0.83 (0.82, 0.84)*	0.72 (0.71, 0.73)*

*Patient IMD*			
*1 = Least deprived*		–	Ref.
*2*		–	0.93 (0.92, 0.95)*
*3*		–	0.90 (0.88, 0.91)*
*4*		–	0.85 (0.83, 0.86)*
*5 = Most deprived*		–	0.75 (0.74, 0.76)*

*Smoking status*			
*Never smoker*		Ref.	Ref.
*Current smoker*		0.86 (0.85, 0.87)*	0.79 (0.78, 0.80)*
*Ex-smoker*		1.06 (1.04, 1.07)*	1.07 (1.07, 1.08)*
*Unknown*		0.60 (0.56, 0.63)*	0.37 (0.36, 0.39)*
*Pregnant*		2.89 (2.83, 2.95)*	–b
*Chronic renal disease*		2.33 (2.20, 2.46)*	–
*Chronic heart disease*		2.01 (1.97, 2.04)*	1.40 (1.38, 1.42)*
*Chronic respiratory disease*		1.47 (1.45, 1.49)*	1.60 (1.58, 1.63)*
*Chronic liver disease*		1.13 (1.10, 1.16)*	–
*Diabetes*		4.25 (4.18, 4.32)*	1.57 (1.54, 1.59)*
*Immunosuppression*		2.36 (2.29, 2.44)*	1.22 (1.17, 1.27)*
*Chronic neurological disease*		2.13 (2.08, 2.18)*	1.10 (1.08, 1.12)*
*Morbidly obese (BMI ≥ 40)*		0.68 (0.67, 0.70)*	0.97 (0.94, 0.99)*
*Unknown*		0.63 (0.62, 0.64)*	0.57 (0.56, 0.58)*
*Composite at-risk score*^c^		2.33 (2.31, 2.36)*	1.39 (1.38, 1.39)*

*GP consultations per year*			
*0–2*		Ref.	Ref.
*3–6*		1.80 (1.79, 1.81)*	1.56 (1.55, 1.57)*
*7+*		2.70 (2.68, 2.72)*	1.97 (1.96, 1.99)*

*Practice region*			
*London*		Ref.	Ref.
*Midlands & East*		1.21 (1.19, 1.23)*	1.32 (1.30, 1.35)*
*North*		1.30 (1.28, 1.32)*	1.42 (1.39, 1.45)*
*South*		1.16 (1.14, 1.18)*	1.21 (1.19, 1.23)*
*Season*^d^		0.97 (0.97, 0.97)*	0.95 (0.95, 0.96)*

*Prior season severity*			
*Low*		Ref.	–
*Moderate*		1.00 (0.99, 1.01)	–
*High*		0.97 (0.96, 0.98)*	–
*Prior season VE (high baseline)*		0.99 (0.98, 0.99)*	0.98 (0.97, 0.98)*

(–) variable was excluded due to lack of significance (p > 0.20), unless noted otherwise.

a We adjusted the odds ratios for all characteristics as listed in the above table (except for the composite at-risk score, where we omitted the individual at-risk conditions to prevent collinearity).

b Pregnancy was excluded from 65+ analysis due to 0 pregnancy records.

c The composite at-risk score was calculated as the patient’s total # of at-risk conditions, excluding age.

d Season was a continuous measure of time (e.g. 1 = 2011, 2 = 2012,… etc.)

* p < 0.05.

### 3.4 Secondary analysis of practices

**Fig. 3:**
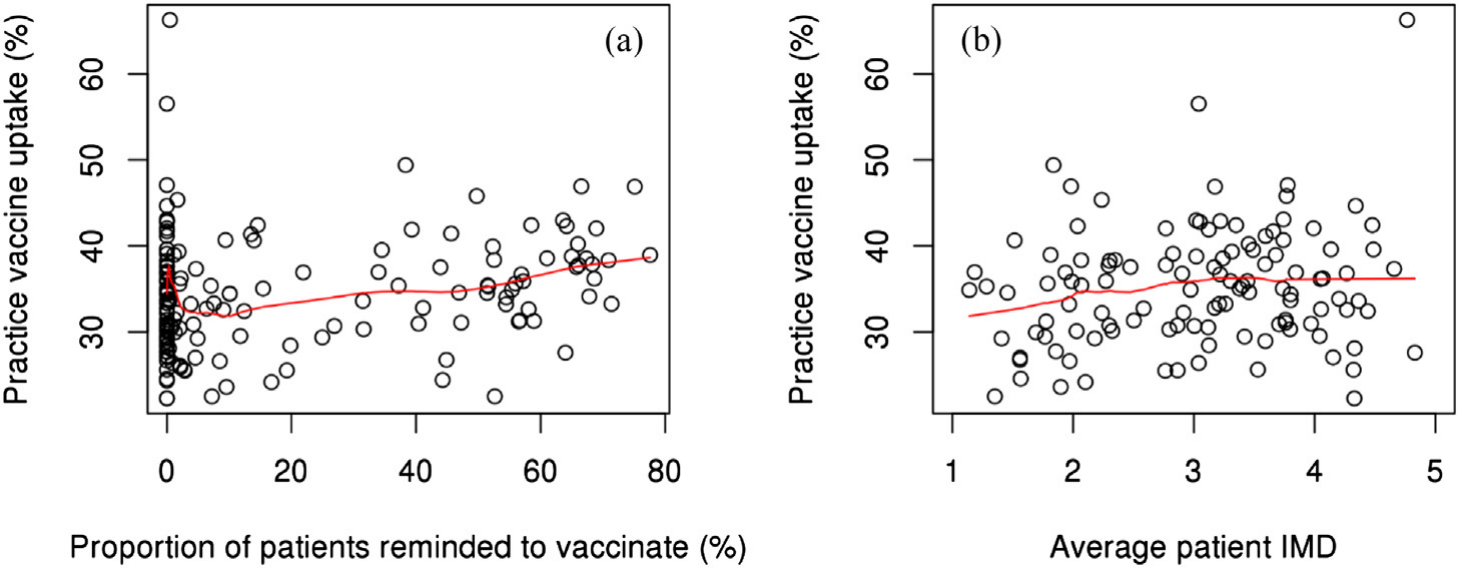
Practice vaccine uptake versus proportion of patients reminded to be vaccinated (a) and average patient IMD (b) among patients 18–64 years old during the 2015–2016 season. Note: Fitted lines were computed using the LOWESS smoothing method.

**Fig. 4:**
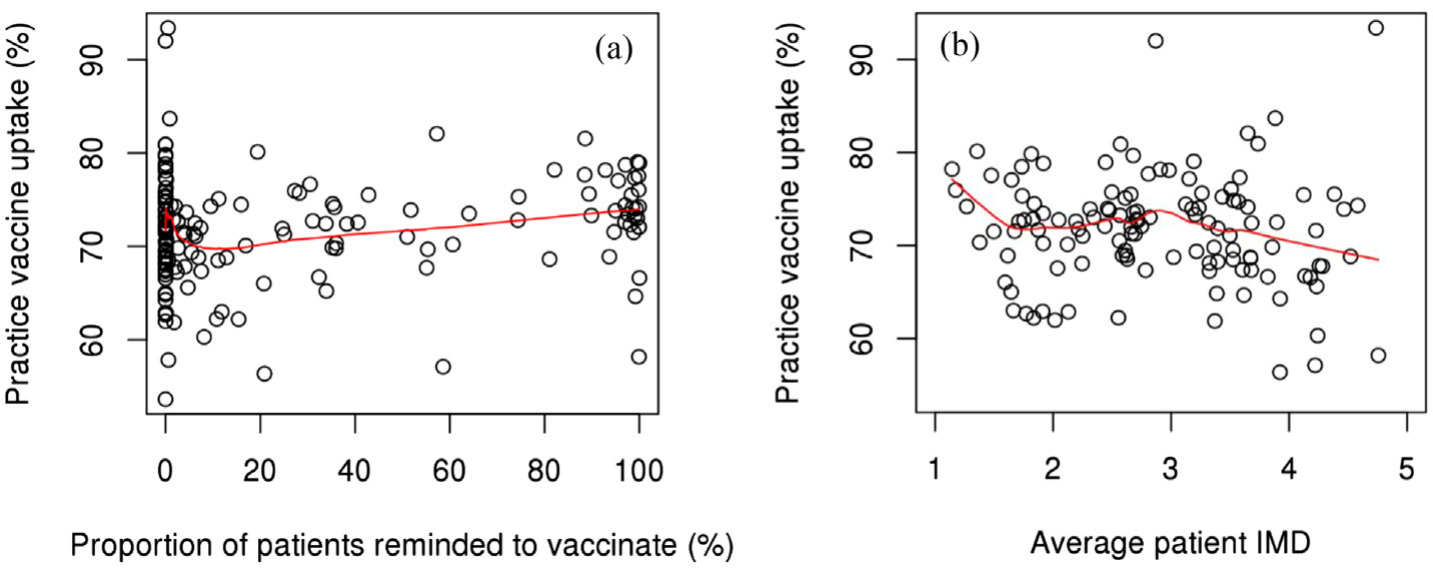
Practice vaccine uptake versus proportion of patients reminded to be vaccinated (a) and average patient IMD (b) among patients 65+ years old during the 2015–2016 season. Note: Fitted lines were computed using the LOWESS smoothing method.

Mean practice uptake varied across quintiles of reminders to be vaccinated, where practices within the 5th quintile had the highest uptake among both age cohorts (Table 5). This trend was not perfectly linear among either age strata though (Figs. 3a and 4a). Mean practice uptake increased as the average patient IMD increased among 18–64 year old patients, while this trend was inverse among 65+ patients (Table 5). The trend among 18–64 year old patients was more subtle, while among 65+ patients it was more substantial, but did not appear to decrease monotonically (Figs. 3b and 4b). Mean practice uptake did not seem to vary in any meaningful pattern across quintiles of IMD – Education or IMD – Income among both age strata (Table 5). Mean practice uptake was however generally higher among practices in areas of greater IMD – Health Deprivation & Disability deprivation, but only so among 18–64 year old patients.

**Table 5:** Mean practice level vaccine uptake percent among patients 18–64 year olds (a) and 65+ years old (b), stratified by quintiles of practice level variables during the 2015–2016 influenza season. Uptake reported as % (SE).

(a) 18–64					
*Variable*	Mean Practice SIV Uptake (%) by Quintile*				
	*1*	*2*	*3*	*4*	*5*
*Reminder to be vaccinated*	34.4 (1.2)	34.2 (1.2)	32.8 (1.2)	34.6 (1.1)	36.8 (1.1)
*Patient IMD*	32.8 (1.2)	34.4 (1.2)	37.5 (1.1)	36.2 (1.1)	35.3 (1.2)
*IMD - Education*	33.2 (1.2)	33.7 (1.2)	33.9 (1.2)	37.5 (1.1)	33.6 (1.1)
*IMD – Health & Disability*	32.3 (1.3)	33.2 (1.1)	35.6 (1.2)	36.3 (1.1)	34.5 (1.1)
*IMD - Income*	32.0 (1.2)	33.0 (1.2)	34.7 (1.1)	36.4 (1.1)	34.6 (1.1)

(b) 65+					

*Variable*	Mean Practice SIV Uptake (%) by Quintile*				

*1*	*2*	*3*	*4*	*5*

*Reminder to be vaccinated*	71.6 (1.5)	71.7 (1.5)	69.8 (1.4)	71.4 (1.4)	73.9 (1.4)
*Patient IMD*	71.9 (1.4)	71.4 (1.5)	74.4 (1.5)	71.2 (1.4)	69.8 (1.4)
*IMD - Education*	73.0 (1.4)	70.7 (1.5)	72.1 (1.5)	72.5 (1.5)	70.2 (1.4)
*IMD – Health & Disability*	71.7 (1.4)	71.1 (1.3)	71.5 (1.5)	71.4 (1.5)	72.7 (1.5)
*IMD - Income*	70.3 (1.3)	73.4 (1.4)	70.9 (1.4)	72.1 (1.6)	71.2 (1.5)

* For IMD, the 5th quintile indicates the greatest level of deprivation. For reminders to be vaccinated, the 5th quintile indicates the greatest proportion of patients reminded to be vaccinated.

## 4 Discussion

Our age-stratified analysis identified several key differences in factors associated with SIV uptake between younger adults (18–64 years) and older adults (65+ years). Odds of SIV uptake increased with age among younger adults, but only increased up to the intermediate age range of 76–85 among older adults, after which it progressively decreased. These findings highlight the non-linear association between age and uptake. Among younger adults, men were less likely to be vaccinated than women; this difference was not observed among older adults.

Disparities in uptake by ethnicity and socioeconomic deprivation were also evident in our analyses. Among both age strata, black patients were consistently less likely than white patients to be vaccinated. Our longitudinal analyses also indicated that Asian patients were less likely to be vaccinated among older adults, but more likely to be vaccinated among younger adults. These trends remained even after adjusting for other factors, highlighting a potential healthcare inequality by ethnicity that should be further investigated [34].

In terms of SES, greater levels of socioeconomic deprivation were associated with decreased odds of being vaccinated, but only so among older adults. Although past UK-based research using pooled analyses has had different conclusions regarding SES [12], our findings are well supported by a more recent age-stratified study of sociodemographic factors of SIV uptake [14]. This discrepancy can likely be explained by methodological and cohort definition differences, as this past research not only pooled patients across age, but also across the UK, including patients from Northern Ireland, Scotland, and Wales.

Nonetheless, this socioeconomic disparity may be indicative of an underlying issue, an access-related barrier to vaccination. In the UK, socioeconomic deprivation has been shown to be associated with lower overall healthcare utilization, in part attributed to access-related barriers among the socioeconomically deprived [35]. Echoing this sentiment, a recent report from the Royal Society for Public Health made a call to action for improved vaccine accessibility, highlighted timing, availability, and location of appointments as three of the primary barriers to adult vaccinations [36]. To address the socioeconomic disparity in uptake among older adults, policy makers may wish to prioritize improving vaccine accessibility, through leveraging the current public health workforce as well as increasing the number of locations where adults can receive the SIV (e.g. pop-up health clinics and workplace vaccinations).

Across the at-risk conditions, morbid obesity was consistently associated with decreased odds of uptake, regardless of age. Although morbidly obese patients are considered at-risk in regards to influenza-related complications, changes in the national influenza vaccine program may explain these findings. In 2015, PHE and the Joint Committee on Vaccination and Immunization (JCVI) made the recommendation to vaccinate morbidly obese patients, concluding that there was a sufficient body of evidence of the benefits of vaccination [37]. However, GPs did not begin receiving payment for vaccination of the morbidly obese under Directed Enhanced Services (DES) until the 2017–2018 influenza season [4]. Furthermore, the association between uptake and morbid obesity that we observed was stronger among younger adults. This may be explained by the lower prevalence of multiple at-risk conditions among younger adults. As it is more likely that a morbidly obese patient under 65 may not have any additional at-risk conditions, relative to morbidly obese patients over 65, these patients would have been ineligible for a reimbursable vaccine during the 2011–2016 influenza seasons.

We found substantial differences in influenza vaccine uptake across practices that remained after controlling for practice area SES measures, and practice urban/rural status. Similar variability has been reported elsewhere, suggesting that the quality of primary care provided by practices may underlie this observed variability [38], [39].

Within practices, it has been previously shown that GPs play a key role in vaccine uptake, where their recommendations are strongly associated with increased uptake [40], [41]. A consultation can serve as an opportunity for a GP to recommend the SIV to a patient, and each subsequent consultation may therefore provide an additional opportunity for such a recommendation to be made. Our findings of greater odds of uptake among those with a greater number of annual GP consultations were consistent with this notion. However, an alternative interpretation of these findings may be that those patients with a higher number of annual consultations are more likely to seek out healthcare in general, and therefore, are more likely to be vaccinated. The dichotomous nature of these two interpretations highlights a fundamental point; while GPs play a key role in improving vaccine uptake, there is also the patient’s inherent propensity for self-care that likely affects their odds of being vaccinated.

Leveraging the longitudinal aspect of the CPRD database, we identified two significant temporal trends in uptake. We have shown that patients of all ages were less likely to be vaccinated following a season with a vaccine of low effectiveness, relative to high effectiveness. Further, patients under 65 were less likely to be vaccinated following a season of high influenza severity, relative to low severity. Within the vaccine hesitancy literature, it has been suggested that vaccine uptake is less likely when one perceives a vaccine to be ineffective, and more likely when one realizes the true severity and associated risks of the disease at hand [42], [43]. While the association we found for prior season VE was consistent with the literature, the observed association with prior season severity was contradictory.

One potential explanation for this discrepancy may be our choice of an indicator for season severity. By using peak influenza-like-illness consultations as a proxy for season severity, we may have inaccurately captured the season severity from the patient’s perspective. Further, the severity of a past season may be too distant in time and memory to influence vaccination behavior, perhaps even overshadowed by the dominating presence of daily consumed mass media, which has been shown to strongly influence utilization of health services [44], [45]. Although we found statistically significant associations with prior season severity and vaccine effectiveness with SIV uptake, these particular findings may not have clinical significance or real-world utility.

One of the primary strengths of our study lies within the type of data used and consequently, the generalizability of the results. With CPRD’s database representing nearly 8% of practices across England, we have captured a wide range of patients in our reference cohort, within and across seasons. The advantages of this are two-fold; our findings are generalizable to a broad group of at-risk adults in England, and we were able to assess various factors over time by continuously following the same patients for up to six years. While the uptake rates reported in our study for older adults aligned closely with those reported by PHE [46], it is worth noting that the rates reported for younger adults in our study were lower than those reported elsewhere. However, this is likely attributable to differences in our definition of the denominator, as we included pregnant or morbidly obese patients without any additional at-risk conditions.

There are some inherent limitations to acknowledge that arise from the use of a primary care database such as the CPRD data. Firstly, we were unable to appropriately account for many other well-known factors associated with uptake, including but not limited to vaccine hesitancy, personal beliefs, social attributes, trust, and peer influence [42]. Although directed surveys can provide an opportunity to collect data on such factors, the associated cost is the introduction of recall, non-response, or selection bias [15], [16]. Further, while some known social determinants of uptake (e.g. marital status, living arrangements, religion, or residing in a long term care home) are recorded in CPRD’s database, we were unable to use them, due to extensive missingness and non-representativeness of the true population values [47]. As for those social determinants we were able to account for, such as ethnicity, we were limited with regard to the level of granularity that we could reliably utilize. While more detailed ethnicity Read codes are available in the CPRD database, missing data and discrepant records ultimately hinder their utility and introduce an additional source of misclassification bias [47].

Similarly, while past SIV uptake behavior is known to be highly predictive of one’s current SIV uptake, we explicitly excluded this predictor from our analysis [9]. Known as a lagged dependent variable (LDV), the use of an outcome from a previous year as a predictor can be highly effective at improving model fit, but consequently, suppresses the explanatory ability of other predictors in the model, while also likely inducing a downward bias in their estimates [48]. The models we have estimated here are intended to provide a nuanced understanding of the overall patterns of SIV uptake within the targeted population, which would have been severely hindered by the inclusion of a LDV. Although a valuable modeling tool under certain circumstances, the use of a LDV simply did not suit the objectives of our study.

Secondly, given that we have used data collected at the practice level, and the recently acquired role of pharmacists in administered vaccinations, it is possible that we may not have captured all records of vaccinations. During the 2013–2014 influenza season, NHS England began allowing pharmacists to administer reimbursable SIV to at-risk patients 13 years or older in the London Region [49]. Two years later, this initiative was then rolled out across the entire nation, enabling all pharmacists to vaccinate any eligible patient [50]. Although pharmacists are required to report all administered vaccines to the patient’s GP, incompleteness of these records has been shown to be a prevalent issue, in part due to incompatibility of IT systems [49]. However, this is unlikely to have been a major hindrance in our analysis, as our uptake rates were similar to those reported by PHE [46].

Thirdly, our findings may have limited generalizability to non-English populations, as the associations we have identified were likely influenced by various England-specific attributes. Taking into consideration country to country differences in SIV recommendations and reimbursements, as well as the heterogeneity of healthcare systems and patient populations, insights obtained from SIV uptake determinants studies tend to be reflective of the region in which they were conducted. However, the methodological framework that we have described and implemented here can be adapted to other regions, to help policy makers assess their respective immunization programs as well as identify potential uptake disparities.

Lastly, there was a progressive decrease in sample size over time across all cohorts that is noteworthy. However, this can be explained by an increasing number of practices dropping out of CPRD enrollment in more recent years. Nonetheless, given the breadth of patients included in our annual cohorts and the considerable number of CPRD-enrolled practices across England that we have captured, our findings remain generalizable to the English at-risk adult population.

## 5 Conclusions

Our age-stratified analysis identified several key differences in factors associated with SIV uptake between at-risk younger adults (18–64 years) and older adults (65+ years). There was evidence of a non-linear association between age and uptake, as well as disparities in uptake by sex, ethnicity, and socioeconomic deprivation between younger adults and older adults. Vaccine uptake in morbidly obese patients was consistently unlikely for all ages. Substantial variation in uptake across practices was also evident, suggesting differential quality of care provided by GPs. Our findings of disparities in uptake warrant further attention by GPs and policymakers alike. Temporally, there was evidence of an association between vaccine uptake and both prior season vaccine effectiveness and prior season severity with odds of uptake. One of the primary strengths of our study lies within the use of a large, validated primary-care database and consequently, the generalizability of these findings to the English at-risk adult population.

## Author contributions

MML conducted all statistical analyses and drafting of the paper. All authors contributed to the protocol development, interpretation of the analyses, critical revisions of the paper, and approved the final paper for submission.

## CRediT authorship contribution statement

**Matthew M. Loiacono:** Conceptualization, Methodology, Software, Formal analysis, Data curation, Writing - original draft. **Salaheddin M. Mahmud:** Conceptualization, Methodology, Writing - review & editing. **Ayman Chit:** Conceptualization, Writing - review & editing, Supervision. **Robertus van Aalst:** Conceptualization, Methodology, Writing - review & editing. **Jeffrey C. Kwong:** Methodology, Writing - review & editing. **Nicholas Mitsakakis:** Methodology, Writing - review & editing. **Luke Skinner:** Conceptualization, Writing - review & editing. **Edward Thommes:** Conceptualization, Methodology, Writing - review & editing. **Hélène Bricout:** Conceptualization, Writing - review & editing. **Paul Grootendorst:** Conceptualization, Methodology, Writing - review & editing, Supervision.

## Declaration of Competing Interest

The authors declare the following financial interests/personal relationships which may be considered as potential competing interests: MML reports financial and non-financial support from Sanofi Pasteur and University of Toronto. HB, AC, LS, ET, and RA are full-time employee of Sanofi Pasteur. SMM has received research grants and/or consulting fees from GlaxoSmithKline, Merck, Sanofi Pasteur, Pfizer and Roche-Assurex. SMM’s work is supported, in part, by funding from the Canada Research Chair Program.
