# Supplementary material for: Patient and practice level factors associated with seasonal influenza vaccine uptake among at-risk adults in England, 2011 to 2016: An age-stratified retrospective cohort study
Source: Vaccine X. 2020 Jan 13;4:100054. doi: 10.1016/j.jvacx.2020.100054 (PMC7011080; doi:10.1016/j.jvacx.2020.100054)
Supplement: Supplementary data 2 [file mmc2.docx]

**Construction of Reference Cohort**

**Enrollment and Disenrollment Dates**

Prior to constructing each annual cohort, we first defined a broader reference study cohort. This cohort identified all patients in CPRD GOLD whom met our inclusion and exclusion criteria. From this reference cohort, annual cohorts were then constructed, where at-risk patients were followed for the duration of the specific year (Example: Season 2011 Cohort = January 1, 2011 to December 31, 2011), based upon their *Enrollment Date* and *Disenrollment Date*.

In order to determine a patient’s entry to (*Enrollment Date*) and exit from (*Disenrollment Date*) the reference study cohort, we defined a series of rules, inclusion, and exclusion criteria, as described below.

For the duration of the study period (*Study Start Date* January 1, 2011 to *Study End Date* December 31, 2016):

*Enrollment Date* was defined as the **latest** of the following:

- *Study Start Date*
- *Date of first registration (frd)*
- *Date of 18th birthday*

An algorithm, based upon the following rules, was used to ensure an accurate *Enrollment Date,* while taking into account the practice up-to-standard (*UTS*)* date:

1. If *UTS* occurs after *Enrollment Date* and before the *Study End Date* and *Disenrollment Date*, then *Enrollment Date* is equal to the *UTS* date
2. If *UTS* occurs after *Study End Date* or *Disenrollment Date*, then *Enrollment Date* is null
3. If *Date of first registration* occurs after *Study End Date* or *Disenrollment Date*, then *Enrollment Date* is null
4. If *Date of 18th birthday* occurs after *Study End Date*, then *Enrollment Date* is null

** Date at which the practice data is deemed to be of research quality; derived by CPRD*

Disenrollment Date was defined as the **earliest** of the following:

- *Study End Date*
- *Death date*
- *Transfer out date*

An algorithm, based upon the following rules, was used to ensure an accurate *Disenrollment Date* and to account for the practice last-collection-date** (*LCD*):

1. If *LCD* occurs prior to *Disenrollment Date* and after *Study Start Date* and after *Enrollment Date,* then *Disenrollment Date* is equal to the *LCD*
2. If *Death date* and/or *Transfer out date* occur prior to *Study Start Date*, then *Disenrollment Date* is null
3. If *Death date* occurs before *Transfer out date* and after the *Study Start Date* and *Enrollment Date*, then *Disenrollment Date* is equal to *Death date*

*** Date of last data collection for the practice*

**Inclusion and Exclusion Criteria**

Once *Enrollment* and *Disenrollment* dates were determined, the finalized reference cohort was created given the following criteria (note: some reiteration is described, based upon the assignment of enroll/disenroll dates):

Inclusion Criteria:

- 18+ years of age before or within *Study Period*
- “Acceptable” patient for research use, as determined by CPRD (accept=1)
- Patient is registered to an England-based practice (practice region ∈ [1,10])
- Patient has at least 1 record in CPRD GOLD, aside from their record in the *Patient* data table
- Patient’s assigned practice is UTS, prior or equal to when a patient enters into the reference cohort (otherwise patient’s entry date is modified accordingly)
- Assigned practice’s last collection date is on or after patient’s exits from the reference cohort (otherwise patient’s exit date is modified accordingly)

Exclusion Criteria:

- *Enrollment* and/or *Disenrollment* date is null
- Gender is unknown or indeterminate
- Coverage from *Enrollment Date* to *Disenrollment Date* is less than one year in duration (e.g. *Disenrollment Date* – *Enrollment date* < 365 days)
- Registration gap > 30 days
- Patient has any record or history of influenza vaccine contraindication or an allergic response

**CPRD’s Measures of Data Quality**

CPRD provides three measures to assess the quality of the data for research purposes: the acceptability of patients, an up-to-standard (UTS) date for practices, and a last-collection-date (LCD) for practices. The acceptable patient indicator identifies any patients with gaps in their registration or instances of poor data recording. Patients who did not meet the acceptability criteria were excluded from our reference cohort. The UTS date indicates the time at which a practice’s data are deemed high enough quality for research, based upon a gap analysis for continuity of data recording and an assessment of death recording. The LCD indicates the most recent date when CPRD had collected data on the practice in which the individual is enrolled [1]. Patient enrollment and disenrollment dates were modified accordingly to account for the practice UTS date and LCD.

References

[1] Herrett, E., et al. Data Resource Profile: Clinical Practice Research Datalink (CPRD). International J Epidemiology 2015;44:827-36. 10.1093/ije/dyv098.
