# Supplementary material for: Patient and practice level factors associated with seasonal influenza vaccine uptake among at-risk adults in England, 2011 to 2016: An age-stratified retrospective cohort study
Source: Vaccine X. 2020 Jan 13;4:100054. doi: 10.1016/j.jvacx.2020.100054 (PMC7011080; doi:10.1016/j.jvacx.2020.100054)
Supplement: Supplementary data 5 [file mmc5.docx]

Table S1. Vaccine uptake (%) per season amongst patients 18-64 years old, overall and stratified by sex, ethnicity, region, and total at-risk conditions.

| *Variable* | | *Season 2011* | *Season 2012* | *Season 2013* | *Season 2014* | *Season 2015* | *Season 2016* |
| --- | --- | --- | --- | --- | --- | --- | --- |
| *Overall* | | 36.0% (153619/426805) | 36.1% (150113/416330) | 35.4% (133107/376169) | 35.0% (110235/314631) | 34.0% (76045/223624) | 33.8% (57338/169544) |
| *Sex* | |  |  |  |  |  |  |
|  | *Male* | 34.8% (72480/208302) | 34.3% (69799/203363) | 33.5% (61731/184036) | 32.7% (50423/154302) | 31.5% (34640/109986) | 31.4% (26165/83411) |
|  | *Female* | 37.1% (81139/218503) | 37.7% (80314/212967) | 37.1% (71376/192133) | 37.3% (59812/160329) | 36.4% (41405/113638) | 36.2% (31173/86133) |
| *Ethnicity* | |  |  |  |  |  |  |
|  | *Asian* | 44.6% (6025/13510) | 45.6% (6413/14079) | 44.7% (5933/13273) | 44.7% (5056/11308) | 44.5% (3587/8063) | 44.6% (3367/7543) |
|  | *Black* | 34.5% (2714/7868) | 34.4% (2930/8527) | 34.7% (2846/8208) | 35.4% (2675/7550) | 33.6% (1721/5124) | 34.4% (1565/4550) |
|  | *Mixed/Other* | 37.6% (31853/84750) | 37.1% (32440/87405) | 36.6% (30697/83984) | 36.0% (26181/72640) | 34.6% (16847/48626) | 33.9% (13381/39485) |
|  | *Unknown* | 32.5% (60371/185500) | 32.4% (55673/171733) | 31.7% (47655/150230) | 31.2% (38204/122310) | 30.4% (26411/86992) | 30.8% (17774/57769) |
|  | *White* | 39.0% (52656/135177) | 39.1% (52657/134586) | 38.2% (45976/120474) | 37.8% (38119/100823) | 36.7% (27479/74819) | 35.3% (21251/60197) |
| *PHE Region* | |  |  |  |  |  |  |
|  | *London* | 35.1% (19723/56270) | 34.6% (20718/59818) | 34.6% (20216/58482) | 33.8% (15222/44969) | 31.2% (10099/32385) | 31.2% (9159/29397) |
|  | *Midlands  & East* | 35.9% (38636/107737) | 36.5% (36291/99484) | 36.3% (29644/81752) | 35.7% (24712/69215) | 35.7% (15117/42370) | 34.9% (12896/36944) |
|  | *North* | 37.7% (34346/91137) | 38.0% (33305/87633) | 36.8% (28989/78780) | 36.2% (21899/60428) | 34.4% (14635/42603) | 35.0% (10615/30297) |
|  | *South* | 35.5% (60914/171661) | 35.3% (59799/169395) | 34.5% (54258/157155) | 34.6% (48402/140019) | 34.1% (36194/106266) | 33.8% (24668/72906) |
| *At-Risk Conditions* | |  |  |  |  |  |  |
|  | *1* | 31.7% (116142/366564) | 31.7% (112941/356472) | 31.0% (99465/321218) | 30.5% (81640/267365) | 29.6% (55838/188953) | 29.3% (41851/142782) |
|  | *2+* | 62.2% (37477/60241) | 62.1% (37172/59858) | 61.2% (33642/54951) | 60.5% (28595/47266) | 58.3% (20207/34671) | 57.9% (15487/26762) |

Note: Numbers in parentheses are (# of vaccines administered /n)

Table S2. Vaccine uptake (%) per season amongst patients 65+ years old, overall and stratified by sex, ethnicity, region, and total at-risk conditions.

| *Variable* | | *Season 2011* | *Season 2012* | *Season 2013* | *Season 2014* | *Season 2015* | *Season 2016* |
| --- | --- | --- | --- | --- | --- | --- | --- |
| *Overall* | | 75.4% (430118/570260) | 74.6% (418477/560772) | 74.4% (382610/514163) | 73.9% (317281/429303) | 71.9% (218946/304658) | 70.8% (150312/212169) |
| *Sex* | |  |  |  |  |  |  |
|  | *Male* | 75.5% (192461/254758) | 74.7% (188256/252044) | 74.4% (172714/232012) | 73.8% (143479/194435) | 71.8% (99146/138107) | 70.9% (68455/96613) |
|  | *Female* | 75.3% (237657/315502) | 74.6% (230221/308728) | 74.4% (209896/282151) | 74.0% (173802/234868) | 71.9% (119800/166551) | 70.8% (81857/115556) |
| *Ethnicity* | |  |  |  |  |  |  |
|  | *Asian* | 77.3% (5377/6953) | 75.0% (5511/7348) | 75.1% (5297/7056) | 74.6% (4340/5821) | 72.9% (3216/4410) | 72.4% (2947/4072) |
|  | *Black* | 66.2% (2481/3750) | 65.1% (2567/3942) | 65.2% (2463/3776) | 64.9% (2026/3120) | 60.8% (1262/2077) | 59.2% (1038/1753) |
|  | *Mixed/Other* | 78.5% (84757/107995) | 76.9% (85275/110923) | 76.8% (83345/108580) | 76.0% (73338/96448) | 73.8% (47225/63995) | 72.0% (35520/49318) |
|  | *Unknown* | 71.9% (187802/261290) | 70.9% (173480/244646) | 70.8% (156451/221037) | 70.4% (125953/178958) | 68.7% (86366/125649) | 68.6% (52547/76628) |
|  | *White* | 78.7% (149701/190272) | 78.2% (151644/193913) | 77.7% (135054/173714) | 77.0% (111624/144956) | 74.5% (80877/108527) | 72.5% (58260/80398) |
| *PHE Region* | |  |  |  |  |  |  |
|  | *London* | 73.3% (46137/62948) | 72.0% (47902/66549) | 72.1% (47409/65731) | 71.0% (35146/49500) | 67.6% (24979/36943) | 64.6% (22281/34466) |
|  | *Midlands  & East* | 75.5% (107470/142427) | 75.4% (100398/133222) | 75.5% (86322/114267) | 74.9% (69868/93293) | 71.9% (40071/55754) | 71.0% (34056/47986) |
|  | *North* | 77.7% (86589/111498) | 76.7% (83558/108957) | 76.2% (75350/98827) | 75.9% (57125/75235) | 74.6% (38303/51378) | 74.5% (25742/34533) |
|  | *South* | 75.0% (189922/253387) | 74.0% (186619/252044) | 73.7% (173529/235338) | 73.4% (155142/211275) | 72.0% (115593/160583) | 71.7% (68233/95184) |
| *At-Risk Conditions* | |  |  |  |  |  |  |
|  | *0** | 67.5% (187444/277893) | 66.1% (180029/272225) | 65.7% (162906/247905) | 64.9% (133278/205354) | 62.5% (90870/145371) | 61.5% (61736/100451) |
|  | *1* | 81.3% (151701/186555) | 80.8% (147938/183121) | 80.5% (135501/168263) | 80.1% (112296/140209) | 78.2% (77578/99182) | 76.9% (53349/69341) |
|  | *2+* | 86.0% (90973/105812) | 85.9% (90510/105426) | 85.9% (84203/97995) | 85.6% (71707/83740) | 84.0% (50498/60105) | 83.1% (35227/42377) |

Note: Numbers in parentheses are (# of vaccines administered /n)

Table S3. SIV uptake (%) across all seasons 2011-2016 for 18-64 adults and 65+ adults, overall and stratified by sex, ethnicity, region, and at-risk conditions.

| *Variable* | | *18-64* | *65+* |
| --- | --- | --- | --- |
| *Overall* | | 35.3% (680457/1927103) | 74.0% (1917744/2591325) |
| *Sex* | |  |  |
|  | *Male* | 33.4% (315238/943400) | 74.0% (864511/1167969) |
|  | *Female* | 37.1% (365219/983703) | 74.0% (1053233/1423356) |
| *Ethnicity* | |  |  |
|  | *Asian* | 44.8% (30381/67776) | 74.8% (26688/35660) |
|  | *Black* | 34.5% (14451/41827) | 64.3% (11837/18418) |
|  | *Mixed/Other* | 36.3% (151399/416890) | 76.2% (409460/537259) |
|  | *Unknown* | 31.8% (246088/774534) | 70.6% (782599/1108208) |
|  | *White* | 38.0% (238138/626076) | 77.1% (687160/891780) |
| *PHE Region* | |  |  |
|  | *London* | 33.8% (95137/281321) | 70.8% (223854/316137) |
|  | *Midlands  & East* | 36.0% (157296/437502) | 74.7% (438185/586949) |
|  | *North* | 36.8% (143789/390878) | 76.3% (366667/480428) |
|  | *South* | 34.8% (284235/817402) | 73.6% (889038/1207811) |
| *At-Risk Conditions* | |  |  |
|  | *Pregnant* | 39.8% (25958/65247) | - |
|  | *Chronic renal disease* | 55.1% (9523/17268) | 83.8% (19590/23373) |
|  | *Chronic heart disease* | 52.7% (116185/220494) | 84.0% (513268/611026) |
|  | *Chronic respiratory disease* | 28.9% (326586/1129437) | 84.0% (356566/424245) |
|  | *Chronic liver disease* | 41.3% (26290/63663) | 79.0% (24445/30955) |
|  | *Diabetes* | 69.5% (200526/288724) | 85.0% (320023/376576) |
|  | *Immunosuppressed* | 48.9% (22908/46879) | 82.4% (37310/45252) |
|  | *Chronic neurological disease* | 51.3% (66483/129695) | 80.9% (257920/318902) |
|  | *Morbid obesity (BMI > 40)* | 27.2% (56780/208900) | 76.5% (38274/50048) |

Note: Numbers in parentheses are (# of vaccines administered/n)

(-) There were no pregnancy records among 65+ patients

Table S4. Unadjusted and adjusted odds ratios (OR) from multivariable cross-sectional analyses amongst patients 18-64 years old during the 2015 to 2016 influenza season.

| *Variable* | | *Season 2015* | |
| --- | --- | --- | --- |
|  |  | *Unadjusted OR (95% CI)* | *Adjusted OR (95% CI) ^a^* |
| *Sex (female)* | | 1.25 (1.23, 1.30)* | 1.09 (1.07, 1.12)* |
| *Age* | |  |  |
|  | *18-25* | Ref. | Ref. |
|  | *26-35* | 1.59 (1.52, 1.70)* | 1.19 (1.13, 1.25)* |
|  | *36-45* | 2.56 (2.45, 2.70)* | 1.78 (1.70, 1.87)* |
|  | *46-55* | 4.46 (4.28, 4.60)* | 2.65 (2.53, 2.78) |
|  | *56-64* | 8.86 (8.50, 9.20)* | 4.31 (4.12, 4.52) |
| *Ethnicity* | |  |  |
|  | *Asian* | 1.32 (1.26, 1.39)* | 1.04 (0.98, 1.10) |
|  | *Black* | 0.94 (0.88, 1.00)* | 0.83 (0.77, 0.89)* |
|  | *Mixed/Other* | 0.91 (0.89, 0.94)* | 0.96 (0.93, 1.00)* |
|  | *Unknown* | 0.70 (0.68, 0.72)* | 0.80 (0.78, 0.82)* |
|  | *White* | Ref. | Ref. |
| *Patient IMD* | |  |  |
|  | *1 = Least deprived* | Ref. | - |
|  | *2* | 1.02 (0.99, 1.10) | - |
|  | *3* | 1.03 (1.00, 1.10) | - |
|  | *4* | 1.01 (0.97, 1.00) | - |
|  | *5 = Most deprived* | 1.02 (0.98, 1.10) | - |
|  | *Unknown* | 0.95 (0.87, 1.00) | - |
| *Smoking status* | |  |  |
|  | *Never smoker* | Ref. | Ref. |
|  | *Current smoker* | 0.81 (0.79, 0.83)* | 0.73 (0.71, 0.75)* |
|  | *Ex-smoker* | 1.42 (1.39, 1.46)* | 1.02 (1.00, 1.05)* |
|  | *Unknown* | 0.15 (0.13, 0.18)* | 0.48 (0.39, 0.58)* |
| *Pregnant* | | 1.57 (1.49, 1.60)* | 3.06 (2.88, 3.25)* |
| *Chronic renal disease* | | 2.20 (2.02, 2.40)* | 2.22 (2.00, 2.46)* |
| *Chronic heart disease* | | 2.14 (2.08, 2.20)* | 1.74 (1.68, 1.80)* |
| *Chronic respiratory disease* | | 0.49 (0.48, 0.50)* | 1.31 (1.28, 1.35)* |
| *Chronic liver disease* | | 1.13 (1.08, 1.20)* | 0.85 (0.81, 0.90)* |
| *Diabetes* | | 5.10 (4.98, 5.20)* | 3.50 (3.39, 3.61)* |
| *Immunosuppression* | | 1.69 (1.60, 1.80)* | 2.21 (2.07, 2.35)* |
| *Chronic neurological disease* | | 2.01 (1.94, 2.10)* | 2.02 (1.94, 2.10)* |
| *Morbidly obese (BMI > 40)* | | 0.72 (0.70, 0.74)* | 0.77 (0.75, 0.80)* |
|  | *Unknown* | 0.19 (0.18, 0.20)* | 0.57 (0.54, 0.59)* |
| *Composite at-risk score ^b^* | | 2.49 (2.44, 2.50)* | 1.91 (1.88, 1.96)* |
| *Consultations per year* | |  |  |
|  | *0-2* | Ref. | Ref. |
|  | *3-6* | 4.10 (3.99, 4.20)* | 3.11 (3.01, 3.20)* |
|  | *7+* | 9.94 (9.67, 10.20)* | 6.07 (5.89, 6.25)* |
| *% of a practice’s patients reminded to vaccinate (10% intervals)* | | 1.02 (1.00, 1.03)* | 1.02 (1.00, 1.04)* |
| *Practice region* | |  |  |
|  | *London* | Ref. | Ref. |
|  | *Midlands & East* | 1.20 (1.05, 1.40)* | 1.32 (1.12, 1.57)* |
|  | *North* | 1.13 (0.99, 1.30) | 1.19 (1.01, 1.40)* |
|  | *South* | 1.12 (1.01, 1.30)* | 1.22 (1.06, 1.40)* |
| *Practice - rural (urban reference)* | | 1.07 (0.93, 1.20) | - |
|  | *Unknown* | 1.05 (0.95, 1.20) | - |
| *Practice IMD - Health* | |  |  |
|  | *1 = Least deprived* | Ref. | - |
|  | *2* | 1.04 (0.89, 1.20) | - |
|  | *3* | 1.16 (1.01, 1.30)* | - |
|  | *4* | 1.20 (1.04, 1.40)* | - |
|  | *5 = Most deprived* | 1.10 (0.95, 1.30) | - |
|  | *Unknown* | 1.15 (1.01, 1.30)* | - |
| *Practice IMD - Income* | |  |  |
|  | *1 = Least deprived* | Ref. | - |
|  | *2* | 1.04 (0.86, 1.20) | - |
|  | *3* | 1.13 (0.94, 1.30) | - |
|  | *4* | 1.21 (1.01, 1.40)* | - |
|  | *5 = Most deprived* | 1.12 (0.93, 1.30) | - |
|  | *Unknown* | 1.16 (0.97, 1.40) | - |
| *Practice IMD - Education* | |  |  |
|  | *1 = Least deprived* | Ref. | - |
|  | *2* | 1.02 (0.87, 1.20) | - |
|  | *3* | 1.03 (0.89, 1.20) | - |
|  | *4* | 1.20 (1.04, 1.40)* | - |
|  | *5 = Most deprived* | 1.02 (0.88, 1.20) | - |
|  | *Unknown* | 1.10 (0.96, 1.30) | - |

^a^ We adjusted the odds ratios for all characteristics as listed in the table (except for the composite at-risk score, where we omitted the individual at-risk conditions to prevent collinearity).

^b^ The composite at-risk score was calculated as the patient’s total # of at-risk conditions.

(-) variable was excluded due to lack of significance (p > 0.20), unless noted otherwise.

* p < 0.05

Table S5. Unadjusted and adjusted odds ratios (OR) from multivariable cross-sectional analyses amongst patients 65+ years old during the 2015 to 2016 influenza season.

| *Variable* | | *Season 2015* | |
| --- | --- | --- | --- |
|  |  | *Unadjusted OR (95% CI)* | *Adjusted OR (95% CI) ^a^* |
| *Sex (female)* | | 1.01 (0.99, 1.02) | 0.98 (0.96, 1.00)* |
| *Age* | |  |  |
|  | *65-70* | Ref. | Ref. |
|  | *71-75* | 1.73 (1.69, 1.80)* | 1.55 (1.52, 1.59)* |
|  | *76-85* | 2.33 (2.29, 2.40)* | 1.83 (1.79, 1.87)* |
|  | *86-95* | 2.19 (2.13, 2.30)* | 1.67 (1.62, 1.72)* |
|  | *96+* | 1.02 (0.93, 1.10) | 1.17 (1.06, 1.29)* |
| *Ethnicity* | |  |  |
|  | *Asian* | 0.96 (0.90, 1.04) | 0.89 (0.83, 0.96)* |
|  | *Black* | 0.64 (0.59, 0.71)* | 0.60 (0.55, 0.67)* |
|  | *Mixed/Other* | 0.94 (0.92, 0.97)* | 0.99 (0.96, 1.02) |
|  | *Unknown* | 0.63 (0.62, 0.65)* | 0.71 (0.69, 0.73)* |
|  | *White* | Ref. | Ref. |
| *Patient IMD* | |  |  |
|  | *1 = Least deprived* | Ref. | Ref. |
|  | *2* | 0.93 (0.90, 0.96)* | 0.91 (0.88, 0.93)* |
|  | *3* | 0.89 (0.86, 0.91)* | 0.85 (0.83, 0.88)* |
|  | *4* | 0.84 (0.82, 0.87)* | 0.80 (0.77, 0.83)* |
|  | *5 = Most deprived* | 0.80 (0.77, 0.83)* | 0.74 (0.71, 0.77)* |
|  | *Unknown* | 0.75 (0.69, 0.82)* | 0.84 (0.76, 0.93)* |
| *Smoking status* | |  |  |
|  | *Never smoker* | Ref. | Ref. |
|  | *Current smoker* | 0.60 (0.58, 0.611)* | 0.64 (0.63, 0.66)* |
|  | *Ex-smoker* | 1.28 (1.26, 1.307)* | 1.11 (1.09, 1.13)* |
|  | *Unknown* | 0.07 (0.06, 0.079)* | 0.28 (0.25, 0.31)* |
| *Pregnant* | | - ^b^ | - ^b^ |
| *Chronic renal disease* | | 1.74 (1.57, 1.90)* | - |
| *Chronic heart disease* | | 2.17 (2.12, 2.20)* | 1.36 (1.33, 1.39)* |
| *Chronic respiratory disease* | | 2.04 (1.99, 2.10)* | 1.53 (1.49, 1.57)* |
| *Chronic liver disease* | | 1.31 (1.22, 1.40)* | - |
| *Diabetes* | | 2.22 (2.16, 2.30)* | 1.48 (1.44, 1.53)* |
| *Immunosuppression* | | 1.64 (1.54, 1.80)* | 1.23 (1.14, 1.31)* |
| *Chronic neurological disease* | | 1.60 (1.56, 1.60)* | 1.06 (1.03, 1.09)* |
| *Morbidly obese (BMI > 40)* | | 1.07 (1.01, 1.13)* | 0.88 (0.83, 0.94)* |
|  | *Unknown* | 0.27 (0.26, 0.28)* | 0.49 (0.47, 0.52)* |
| *Composite at-risk score ^c^* | | *1.79 (1.77, 1.80)** | 1.33 (1.32, 1.35)* |
| *Consultations per year* | |  |  |
|  | *0-2* | Ref. | Ref. |
|  | *3-6* | 3.39 (3.31, 3.50)* | 2.73 (2.67, 2.80)* |
|  | *7+* | 6.62 (6.48, 6.80)* | 4.41 (4.30, 4.51)* |
| *% of a practice’s patients reminded to vaccinate (10% intervals)* | | 1.01 (1.00, 1.02)* | 1.02 (1.01, 1.03)* |
| *Practice region* | |  |  |
|  | *London* | Ref. | Ref. |
|  | *Midlands & East* | 1.24 (1.08, 1.40)* | 1.30 (1.11, 1.53)* |
|  | *North* | 1.40 (1.23, 1.60)* | 1.51 (1.29, 1.76)* |
|  | *South* | 1.21 (1.08, 1.30)* | 1.32 (1.15, 1.51)* |
| *Practice - rural (urban reference)* | | 0.95 (0.83, 1.10) | - |
|  | *Unknown* | 1.02 (0.92, 1.10) | - |
| *Practice IMD - Health* | |  |  |
|  | *1 = Least deprived* | Ref. | - |
|  | *2* | 0.97 (0.83, 1.10) | - |
|  | *3* | 0.99 (0.85, 1.20) | - |
|  | *4* | 0.99 (0.85, 1.20) | - |
|  | *5 = Most deprived* | 1.05 (0.90, 1.20) | - |
|  | *Unknown* | 1.03 (0.89, 1.20) | - |
| *Practice IMD - Income* | |  |  |
|  | *1 = Least deprived* | Ref. | - |
|  | *2* | 1.16 (0.96, 1.40) | - |
|  | *3* | 1.02 (0.85, 1.20) | - |
|  | *4* | 1.09 (0.91, 1.30) | - |
|  | *5 = Most deprived* | 1.04 (0.87, 1.30) | - |
|  | *Unknown* | 1.10 (0.91, 1.30) | - |
| *Practice IMD - Education* | |  |  |
|  | *1 = Least deprived* | Ref. | - |
|  | *2* | 0.89 (0.75, 1.10) | - |
|  | *3* | 0.95 (0.82, 1.10) | - |
|  | *4* | 0.98 (0.85, 1.10) | - |
|  | *5 = Most deprived* | 0.87 (0.75, 1.00) | - |
|  | *Unknown* | 0.96 (0.83, 1.10) | - |

^a^ We adjusted the odds ratios for all characteristics as listed in the table (except for the composite at-risk score, where we omitted the individual at-risk conditions to prevent collinearity).

^b^ Pregnancy was excluded from 65+ analysis due to 0 pregnancy records.

^c^ The composite at-risk score was calculated as the patient’s total # of at-risk conditions.

(-) variable was excluded due to lack of significance (p > 0.20), unless noted otherwise.

* p < 0.05
