# Supplementary material for: Patient and practice level factors associated with seasonal influenza vaccine uptake among at-risk adults in England, 2011 to 2016: An age-stratified retrospective cohort study
Source: Vaccine X. 2020 Jan 13;4:100054. doi: 10.1016/j.jvacx.2020.100054 (PMC7011080; doi:10.1016/j.jvacx.2020.100054)
Supplement: Supplementary data 6 [file mmc6.docx]

**Statistical Analysis Models**

**Cross-Sectional Analyses**

*Mixed-Effects Logistic Regression Model (Random Intercept)*

SIV uptake for the $i^{th}$patient $\left( i=1,2,\ldots n \right)$ registered to the $j^{th}$ practice $\left( j=1,2,\ldots m \right)$, given patient characteristics, practice characteristics, and the random effect of the practice can be modeled as such:

$${SIV}_{ij} |\pi_{ij} \sim Bernoulli(\pi_{ij})$$

$$\pi_{ij}=P\left( {SIV}_{ij}=1 \right| X1,X2,\gamma_{j})$$

$$logit\left( \pi_{ij} \right)=\gamma_{j}+{X1}_{i}\beta_{1}+{X2}_{j}\beta_{2}+e_{ij}$$

$$\gamma_{j}\sim N(0,\sigma_{u}^{2})$$

$$e_{ij}\sim N(0,\sigma_{e}^{2})$$

Where:

${SIV}_{ij}$=1 if the $i^{th}$ patient registered to the $j^{th}$ practice received an SIV, = 0 otherwise

$\pi_{ij}$ refers to the probability of SIV uptake for the$i^{th}$ patient registered to the $j^{th}$ practice, conditional upon all patient and practice characteristics in the model, as well as the random effect $\gamma_{j}$

$\gamma_{j}$ refers to the random effect (intercept) of the $j^{th}$ practice

$X1$ refers to an $n x p_{1}$ matrix of patient predictors

$\beta_{1}$ refers to a $p_{1} x 1$ column vector of parameters to be estimated

$X2$ refers to an $n x p_{2}$ matrix of predictors of the patient’s registered practice

$\beta_{2}$ refers to a $p_{2} x 1$ column vector of parameters to be estimated

$e_{ij}$ refers to the error term

$\sigma_{u}^{2}$ refers to the variance of the normal distribution of the random effect $\gamma_{j}$

$\sigma_{e}^{2}$ refers to the variance of the normal distribution of the error term $e_{ij}$

**Longitudinal Analyses**

*General Estimating Equation (GEE) Logistic Regression Model* ^[[1]](#footnote-1)^

SIV uptake for the $i^{th}$patient $\left( i=1,2,\ldots m \right)$ in the $t^{th}$ time period $\left( t=1,2,\ldots n_{i} \right)$ , where there are $n_{i}$ observations for each patient $i$ and a total of $N=m*n_{i}$ observations, can be modeled as such:

$${SIV}_{it} |\pi_{it} \sim Bernoulli(\pi_{it})$$

$$\pi_{it}=P\left( {SIV}_{it}=1 \right| X)=E\left( {SIV}_{it} \right| X)$$

$$logit\left( \pi_{it} \right)=\beta_{0}+{X1}_{it}\beta_{1}+{X2}_{t}\beta_{2}+T\beta_{3}+e_{it}$$

$$e_{it}\sim N(0,\sigma_{e}^{2})$$

Where:

${SIV}_{it} {SIV}_{ij}$=1 if the $i^{th}$ patient received an SIV in the $t^{th}$ time period, = 0 otherwise

$\pi_{it}$ refers to the probability of SIV uptake for the$i^{th}$patient in the $t^{th}$ time period, conditional upon all patient and season characteristics in the model

$X1$ refers to an $N x p_{1}$ matrix of patient predictors

$\beta_{1}$ refers to a $p_{1} x 1$ column vector of parameters to be estimated

$X_{2}$ refers to an $N x p_{2}$ matrix of season predictors

$\beta_{2}$ refers to a $p_{2} x 1$ column vector of parameters to be estimated

$t$ refers to the time period such that $t=1$ is the 2011 season, $t=2$ is the 2012 season, and so on

$\beta_{3}$ refers to the estimated coefficient for time

$e_{it}$ refers to the error term

$\sigma_{e}^{2}$ refers to the variance of the normal distribution of the error term $e_{it}$

1. Robust standard errors were estimated. Clustering was specified at the patient level due to repeated observations for each patient. An exchangeable working correlation matrix structure was specified. [↑](#footnote-ref-1)
